# Antithrombotic and Antiatherosclerotic Properties of Olive Oil and Olive Pomace Polar Extracts in Rabbits

**DOI:** 10.1155/2007/36204

**Published:** 2007-07-25

**Authors:** Nektaria Tsantila, Haralabos C. Karantonis, Despina N. Perrea, Stamatios E. Theocharis, Dimitrios G. Iliopoulos, Smaragdi Antonopoulou, Constantinos A. Demopoulos

**Affiliations:** ^1^Laboratory of Biochemistry, Faculty of Chemistry, School of Sciences, National and Kapodistrian University of Athens, Athens 15771, Greece; ^2^Department of Science of Nutrition-Dietetics, Harokopio University, Athens 17671, Greece; ^3^Laboratory of Experimental Surgery and Surgical Research, School of Medicine, National and Kapodistrian University of Athens, Athens 11527, Greece; ^4^Department of Forensic Medicine and Toxicology, School of Medicine, National and Kapodistrian University of Athens, Athens 11527, Greece

## Abstract

Olive oil polar lipid (OOPL) extract has been reported to inhibit atherosclerosis development on rabbits. Olive pomace polar lipid (PPL) extract inhibits PAF activity in vitro and the most potent antagonist has been identified as a glycerylether-sn-2-acetyl glycolipid with common structural characteristics with the respective potent antagonist of OOPL. The aim of this study was to investigate the effect of PPL on early atherosclerosis development on rabbits and to compare it with the antiatherosclerotic effect of OOPL. OOPL and PPL inhibition potency, towards both PAF action and PAF binding, was tested in vitro on washed rabbit platelets. Consequently, rabbits were divided into three groups (A, B, and C). All groups were fed atherogenic diet for 22 days. Atherogenic diets in groups B and C were enriched with OOPL and PPL, respectively. At the end of the experimental time, rabbits were euthanized and aortic samples were examined histopathologically. OOPL and PPL inhibited PAF-induced aggregation, as well as specific PAF binding, with PPL being more potent. Free and bound PAF levels and PAF-AH activity were significantly elevated at the end of the experimental time. Plasma total cholesterol, HDL cholesterol, LDL cholesterol, and triglycerides levels were also found increased. Groups B and C exhibited significantly increased values of EC_50_ compared to group A. Histopathological examination revealed that the development of early atherosclerosis lesions in groups B and C were significantly inhibited compared to group A. Significant differences were noted in the early atherosclerosis lesions between groups B and C, thus indicating that PPL exhibit its anti-atherosclerotic activity by blocking PAF receptor. Specific PAF antagonists with similar in vitro and in vivo bioactivity to those that have been previously reported in OOPL exist in PPL.

## 1. INTRODUCTION

Coronary heart disease (CHD) is the major source of mortality in the Western World. Although it was believed that this is principally a problem of the developed world, new evidence, provided by the World Health Organization, shows that cardiovascular disease will become a major problem in developing countries as well, unless they stop acquiring Western nutritional habits. Heart attack and stroke are the clinical results of a systemic vascular process widely known as atherosclerosis [1]. Atherosclerosis can be described as a systemic vascular process consisting microscopically of foam cell formation in the subendothelial space, inevitably leading to fatty streak and then fibrous plaque formation [2].

The importance of Mediterranean diet on preventing CHD has been indicated by several studies [3–5]. Olive oil is the main source of fat in the aforementioned diet, thus its beneficiary effects on health have been widely examined, both in vitro and in vivo [4–7].

Olive oil is extracted from olive fruits by pressure, thus resulting into two wastes: olive mill wastewater (OMW) and olive pomace (P) [8, 9]. Olive pomace or just pomace still retains a small amount of olive oil and mainly consists of water, olive skin, olive flesh, and pit fragments; therefore, its major ingredients are sugars (mainly polysaccharides), proteins, fatty acids (oleic acid and other C2–C7 fatty acids), polyalcohols, polyphenols, and other pigments [9–12]. It is mainly used for extracting olive pomace oil (secondary value oil) [12, 13]; however, it can be used as a fuel as well [14].

According to our theory, platelet activating factor (PAF) is implicated in atherogenesis [2] and this is also supported by other researchers [15–18]. The increase of PAF levels and activity in blood, which occurs both in pathological inflammatory conditions and during oxidative stress through LDL and membrane phospholipid oxidation, is believed to be of crucial importance for the initiation of atherosclerosis. PAF activity can be controlled either by PAF-AH or by PAF antagonists derived either from food or produced endogenous. Absence of such antagonists can result to uncontrolled PAF activity [2]. Such PAF antagonists have been isolated from various food stuffs like yogurt [19], honey [20], wine [21–23], fish [24, 25], and olive oil [4, 26], which constitute food stuffs of the Mediterranean diet, thus enhancing our understanding of this diet's beneficial effects on health.

Our group has previously reported the inhibitory effect of olive oil on experimental atherosclerosis induction in rabbits. It was, also, demonstrated that this inhibitory effect is mainly attributed to OOPL, mainly containing PAF antagonists, rather than to olive oil neutral lipids (OONLs), mainly containing oleic acid and antioxidants [27].

Since, on one hand, a percentage of olive oil is still retained in olive pomace [12] and on the other hand olive pomace, as a waste of olive oil industry, has low interest value, the existence of PAF antagonists in olive pomace, could contribute to increase its biological value. The existence of PAF antagonists in olive pomace polar lipid (PPL) extract has already been reported and the bioactive compound has been chemically characterized as a glycerylether-sn-2-acetyl glycolipid sharing structural features with the respective one of OOPL [28]. The aim of this study is to examine in vivo the antithrombotic and antiatherosclerotic activity of PPL on cholesterol fed rabbits and to compare it with the effect of OOPL.

## 2. MATERIALS AND METHODS

### 2.1. Materials

Solvents of analytical grade and for high-performance liquid chromatography (HPLC) and gas chromatography (GC) analyses, as well as silicic acid, 35–70 meshes, ASTM 7733 for column chromatography, were supplied from Merck (Darmstadt, Germany). Bovine serum albumin (BSA), PAF (1-O-hexadecyl-2-acetyl-sn-glycero-3-phosphocholine), lipid standards for HPLC and GC, trichloroacetic acid (TCA), cholesterol (∼95% (GC), equivalent to USP/NF), naphthalene (scintillation grade), and histopaque −1077 were obtained from Sigma (St. Louis, Mont, USA). [^3^H]-acetyl PAF (10 Ci/mmol) (NET 910) was purchased from NEN (Dupont, Boston, Mass, USA). 2,5-diphenyloxazole (PPO) and 1,4-bis(5-phenyl-2-oxazolyl) benzene (POPOP) were purchased from BDH Chemicals (Dorset, England). Scintillation liquid cocktail (dioxane base) was prepared by diluting 7 g PPO, 0.3 g POPOP, and 100 g napthalene in 200 mL H_2_O and then transferred to 1 L of dioxane. Tris buffer, pH 7.4, contained 50 mM Tris. The anticoagulant solution contained 0.065 M citric acid and 0.085 M sodium citrate. Virgin olive oil of Koroneiki variety extracted by pressure and its respective olive pomace came from Kalamata, Greece.

### 2.2. Methods

#### 2.2.1. Modified counter current distribution

Olive oil and pomace were subjected to a modified counter current distribution [4]. According to this method, the ethanol phase at the end yields 96% of the polar lipids as evaluated with [^3^H]-PAF, whereas the combined petroleum ether phases contain the neutral lipid class. This modified counter current distribution is normally applied in vegetable oils [4, 29]. Briefly, every 100 mL of olive oil were placed in a separatory funnel and diluted in 400 mL of petroleum ether (PE) solvent, thus constituting the initial total volume of 500 mL. This solution was then mixed with 100 mL of ethanol 87% solvent. After 5 minutes, the two phases were separated and a new amount of 100 mL ethanol solvent was added in the equilibrated upper phase of PE extract, followed by the equilibration and separation of the two phases. The whole procedure was repeated three times totally, thus obtaining at the end the OOPL in ethanol extracts of final volume 300 mL.

**Figure 1:**
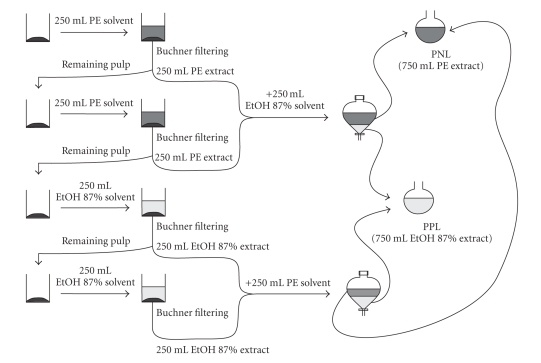
Schematic representation of polar lipid extraction from olive pomace, abbreviated as PPL in the text. PNL: pomace neutral lipids.

A modified extraction (Figure 1) of the aforementioned extraction procedure was applied on olive pomace. Briefly, for every 100 g of olive pomace, 250 mL of PE solvent were added. After 5 minutes in stirring, the liquid is separated from olive pomace through Buchner filtering. The procedure was repeated once more in the remaining olive pomace thus giving 500 mL of PE extract in total, which were placed in a funnel containing 250 mL of ethanol solvent. The two phases were equilibrated and then separated. In the remaining residue of olive pomace, 250 mL of ethanol solvent were added twice and after 5 minutes in stirring, both liquids were removed through Buchner filtering. Ethanol extracts (500 mL) were then placed in a funnel containing 250 mL of PE solvent and the two phases were separated after equilibration. All ethanol phases, of 750 mL total volume, were pooled together, thus consisting the polar lipid extract of olive pomace (PPL), while all PE phases, also 750 mL total volume, consisted the neutral lipid extract, which was not further used.

The evaluation of the above method concerning the recovery of polar lipids from olive pomace was performed with [^3^H]-PAF.

Petroleum ether (b.p. 40–70°C) and ethanol 87% solvents were preequilibrated with each other, prior use in extraction procedures.

#### 2.2.2. Preparation of washed rabbit platelets

Washed rabbit platelets were prepared as previously described [30].

#### 2.2.3. Biological assay on washed rabbit platelets

PAF was dissolved in 2.5 mg of BSA per 1 mL of saline. The examined samples were dissolved in absolute ethanol and BSA 2.5 mg/mL saline was added to give a ratio of ethanol/BSA 1/19. The platelet aggregation induced by PAF (2.5 × 10^−11^ M, final concentration) was measured as PAF-induced aggregation, in washed rabbit platelets, before (considered as 0% inhibition) and after the addition of various concentrations of the examined sample as previously described [30]. Consequently, the plot of percent inhibition (ranging from 0 to 100%) versus different concentrations of the sample is linear. From this curve, the concentration of the sample that inhibited 50% PAF-induced aggregation was calculated, and this value was defined as IC_50_. Buffer control data were performed with 2.5 mg of BSA per 1 mL saline and the results were appropriately corrected. In addition, various concentrations of each examined sample were added into the aggregometer cuvette. According to cross-desensitization experiments that were performed, platelets were desensitized by the addition of the examined lipid at a concentration that caused reversible aggregation. Second stimulation with PAF was performed immediately after complete disaggregation. The study was performed using a Chronolog aggregometer (model 400) (Havertown, Pa, USA) coupled to a Chronolog recorder at 37°C with constant stirring at 1200 rpm.

#### 2.2.4. [^3^H]-PAF binding to washed rabbit platelets

The experiments involving the specific binding of [^3^H]-PAF to washed rabbit platelets and its inhibition by OOPL and PPL were performed as previously described with some modifications [31]. Briefly, [^3^H]-PAF (1 nM), PAF (1 *μ*M), OOPL, and PPL were diluted in absolute ethanol, and BSA 10 mg/mL in Tg-Ca^2+^ buffer pH 7.2 was added to give a ratio of ethanol/BSA 1/19. Incubations were carried out in a total 0.5 mL volume. In 360 *μ*L, Tg buffer pH 7.2 contained CaCl_2_ 1.72 mM, [^3^H]-PAF (1 nM) in the presence or absence of either PAF (1 *μ*M), OOPL, or PPL were added and the volume was adjusted in 460 *μ*L with BSA 10 mg/mL in Tg-Ca^2+^ buffer pH 7.2. Then, 40 *μ*L of washed rabbit platelets (1 × 10^8^ platelets) in Tg buffer pH 6.2 were added to start the incubation time. Double incubations were carried out at 22°C for 4 minutes, a time period where [^3^H]-PAF degradation was calculated to be less than 1%. Termination was performed with the addition of 0.7 mL ice-cold Tg-Ca^2+^ buffer pH 7.2, followed by rapid centrifugation at 11500 × g for 2 minutes at 4°C. The specific PAF antagonist, BN 52021, was used as a reference compound. The radioactivity was measured by scintillation counting on a 1209 Rackbeta liquid scintillation counter from Pharmacia (Wallac, Finland). Nonspecific binding was defined as the total binding measured in the presence of excess unlabelled PAF (1 *μ*M) and specific binding was defined as the difference between total binding and nonspecific binding. The percent inhibition was expressed as %I = (total binding − total binding with tested compound)/specific binding × 100. The IC_50_ value was defined as the concentration of inhibitor required obtaining 50% inhibition of the PAF specific binding. Scatchard analysis was performed with GraphPad Prism software (San Diego, Calif, USA).

#### 2.2.5. Chemical determinations

Phosphorus determination was carried out according to Bartlett [32]. Sugar determination was carried out as referred by Galanos and Kapoulas [33]. Phenol content determination was performed using a modified method of Singleton and Rossi [34]. Briefly, samples were dried under a stream of nitrogen and dissolved in 3.5 mL of water. An amount of 0.1 mL Folin-Ciocalteu reagent was added, followed after 3 minutes by addition of 0.4 mL of 35% aqueous Na_2_CO_3_. The reaction mixture was kept for 1 hour, and the intensity of the blue color was measured at 725 nm. Standards of gallic acid were prepared similarly. Ester determination was carried out according to the method of Renkonen [35].

#### 2.2.6. Fatty acids analysis

GC analysis was carried out on a Shimadzu model GC-17A gas chromatograph (Kyoto, Japan), using an Agilent J & W DB-23 capillary column (60 m × 0.25 mm, i.e., 0.25 *μ*m) purchased by Agilent Technologies (Santa Clara, Calif, USA).

Fatty acids contained in both polar lipid extracts were quantified by GC analysis. Both samples were dried under a stream of nitrogen and then diluted in 1 mL of hexane. Methyl-esterification was performed by the addition of 4 mL of KOH 0.5 N in methanol/water (90/10) at room temperature for 5 minutes under continuous vortexing. After that the solution was neutralized by the addition of HCl 6N and 2 mL of H_2_O were added and the fatty acid methylesters were extracted twice by 2 mL hexane each time. Moisture from the hexane extract was removed using Na_2_SO_4_.

Oven thermal program started at 120°C and remained at this temperature for 5 minutes, then rose at 180°C within 6 minutes, followed by a temperature increase at 220°C within 2 minutes. The temperature remained at 220°C for 25 minutes. Inlet and detector temperatures were 220°C and 230°C, respectively [36].

#### 2.2.7. Animal handling and treatment

Eighteen healthy male New Zealand rabbits of specific weight and age were purchased from a commercial breeder and were individually housed in atomic stainless steel cages in constant conditions of temperature (19 + 1°C), relative moisture (55 + 5%), and air conditioning (12 full changes of air per 1 hour). The light/darkness ratio was 12 hour/12 hour. Rabbits were acclimatized for 5 days before the beginning of the study. Living conditions and animal handling were according to the European Regulation 609/86. The local veterinary authorities and animal ethics committee approved the study.

Rabbits were randomly divided into three groups of six animals each and were given specific diet for 22 days. Group A was given atherogenic diet, while groups B and C were given atherogenic diet enriched with OOPL (0.16% w/w) and PPL (0.13% w/w), respectively. Their food was prepared freshly every three days before consumption.

On the 22nd day, rabbits were given xylazine (Rompun, Bayer, Leverkusen, Germany) 5 mg/Kg body weight and cetamine (Fort Dodge Laboratories Inc., Fort Dodge, Iowa, USA) 25 mg/Kg body weight intramuscularly. Rabbits fell unconscious and soon afterwards euthanasia took place by injecting Pentothal (Hospital Products Division, Abbott Laboratories Abbott Park, Ill, USA) 20 mg/Kg body weight, intravenously. Through a median longitudinal incision, the thoracic and peritoneal cavities were opened and the aorta was dissected from the aortic valve down to the aortic bifurcation.

#### 2.2.8. Biochemical measurements

At the beginning (0 days) and at the end of the experimental time (22 days), blood was collected from all rabbits through the main ear artery and was placed in polyethylene tubes containing anticoagulant with a ratio of blood/anticoagulant 9 : 1 (v/v).

##### 2.2.8.1. PRP aggregation

Platelet-rich plasma (PRP) was obtained by centrifugation of blood samples at 562 × g for 13 minutes, while platelet-poor plasma (PPP) was obtained by further centrifuging the specimens at 1750 × g for 20 minutes. The centrifugation was performed on Heraeus Labofuge 400R (Hanau, Germany) at 24°C. PRP concentration was adjusted to 300 000 platelets/mL using the respective PPP.

Aliquots of PAF solution in chloroform/methanol (1 : 1 v/v) were evaporated under a stream of nitrogen, and were redissolved in BSA (0.25% in saline) in order to obtain PAF solutions with final concentrations ranging from 1.0 × 10^−8^ to 1.0 × 10^−5^ M. The maximum reversible or the minimum irreversible PAF-induced platelet aggregation was determined as the 100% aggregation, and then various PAF concentrations were added, so as to achieve aggregations between 20% and 80%. These PAF-induced aggregations were of linear response to the respective PAF concentration; therefore, the EC_50_ value was calculated. EC_50_ accounts for the PAF concentration inducing 50% aggregation. These studies were performed using a Chronolog aggregometer (model 400) coupled to a Chronolog recorder at 37°C with constant stirring at 1200 rpm [32].

##### 2.2.8.2. Determination of PAF-AH activity

PAF-AH activity was determined as previously described [37]. Briefly an amount of PAF was dissolved in chloroform/methanol (1 : 1; v/v) and mixed with an appropriate amount of [^3^H]-acetyl-PAF. The mixture was dried under a stream of nitrogen and redissolved in BSA (10 *μ*g/*μ*L in Tris-HCl buffer pH 7.4), giving the [^3^H]-acetyl PAF solution with a final concentration of 800 *μ*M and a specific activity of 2500 cpm/nmol that was used as the substrate of PAF-AH. PPP was then placed in Tris-HCl 50 mM pH 7.4 with a ratio of 3/2000 at 200 *μ*L final volume. The whole mixture was incubated at 37°C for 2 minutes and the reaction was initiated by adding 5 *μ*L of 800 *μ*M [^3^H]-acetyl PAF/PAF solution in BSA (10 *μ*g/*μ*L in Tris-HCl buffer pH 7.4). The reaction took place at 37°C for 15 minutes and stopped by binding the unreacted [^3^H]-acetyl PAF with an excess of BSA solution (0.75 mg/mL, final concentration) following by precipitation with TCA with a final concentration of 9.6% (v/v). The mixture was placed in ice bath for 15 minutes and then was centrifuged at 16,000 × g for 5 minutes, at 4°C. The [^3^H]-acetate released from the reaction was measured on a liquid scintillation counter (1209 Rackbeta, Pharmacia) by placing 0.1 mL of the suspension liquid into 5.0 mL of scintillation liquid cocktail (dioxane base). Blank assay was performed with no added plasma. The enzyme activity was expressed as nmol of PAF degraded per minute per *μ*L plasma.

##### 2.2.8.3. Determination of PAF levels in rabbit blood

Determination of free and bound PAF levels was carried out according to Demopoulos et al. [38]. Briefly, 5 mL of blood was collected into glass centrifuge tubes containing 20 mL of cold absolute ethanol. The whole is centrifuged at 280 × g for 20 minutes, at 4°C. The supernatant liquid (containing free PAF levels) was then separated from the cellular pellet (bound PAF levels) and both samples were subjected to extraction according to the Bligh-Dyer method [39]. The total lipid fraction from both samples was then further separated into lipid classes using glass column liquid chromatography (20 cm × 1 mm i.d.). The lipid class containing the polar lipid class (including PAF) was then purified through HPLC using a cation exchange column, SCX Partisil 10 *μ*m 25 × 4.6 cm i.d. purchased by Whatman (Maidstone, UK) at room temperature, where PAF was collected at approximately 30 minutes [40]. The exact PAF concentration in the specimen was then measured through biological assay on washed rabbit platelets, where the induced aggregation versus known synthetic PAF concentrations is linear [30]. The analysis was performed on a Hewlett-Packard series 1100 (Avondale, PA, USA), equipped with a 100 *μ*L loop Rheodyne (i 7725) injector. An 1100 HP UV spectrometer was used as detector at 208 nm. The spectrometer was connected to a Hewlett-Packard (Avondale, PA, USA) model HP-3396A integrator plotter. All necessary centrifugations were performed on Heraeus Labofuge 400R (Hanau, Germany).

##### 2.2.8.4. Lipid profile

Plasma total cholesterol (TC), LDL cholesterol (LDL-C), HDL cholesterol (HDL-C), and triglyceride (TG) concentrations were measured by standard commercial enzymatic methods (bioMerieux, Lyon, France) using a parallel-multichannel analyzer (Type 7170A, Hitachi, Tokyo, Japan).

#### 2.2.9. Histopathological examination

Thoracic aorta specimens were fixed in 10% buffered formaldehyde solution, sectioned and embedded in paraffin wax using conventional techniques. For the histopathological examination 5 *μ*m thickness tissue slide sections were then cut, transferred on slides and stained with haematoxylin and eosin (H-E).

#### 2.2.10. Atherosclerosis evaluation

Conventional measurements of early atherosclerosis lesions in the histopathological tissue sections of resected aortas were performed, using an automated image analysis system. The apparatus comprised a Sony-Exwave HAD Color Video Camera (Sony Corporation, Japan), fitted to a Zeiss Axiostar light microscope (Zeiss, Germany), a host computer (Pentium 90 MHz, 32 MB RAM) and a Sigmascan version 2.0 image analysis software (Jandel Scientific, Erkrath, Germany). Foam cell formation is characteristic of the early atherosclerosis lesions. In the present study, early atherosclerosis lesions were observed as foam cell layers developed inside the blood vessels. Thickness and surface area of the lesions were assessed as previously described [7].

#### 2.2.11. Statistical analysis

Normality tests were applied using the Kolmogorov-Smirnov criterion. Results from ex vivo experiments were expressed as median (25th percentile-75th percentile). The Mann-Whitney *U*-test was performed to assess differences among different groups. The Wilcoxon sign test was used to assess differences in the same group at different time intervals. Results from in vivo experiments concerning food consumption, rabbit weight, and body weight increase in all three groups as well as IC_50_ values and were expressed as mean ± standard deviation (sd). The *t*-test was performed to assess differences between IC_50_ values, food consumption, rabbit weight, body weight increase, and thickness and surface of early atherosclerosis lesions among all three groups. Differences were considered to be statistically significant when *P* < .05. Data were analyzed using a statistical software package (SPSS for Windows, 10.0.1, 1999, SPSS Inc., Chicago, Ill, USA).

## 3. RESULTS

Modified counter current distribution was used as extraction method in order to obtain the two lipid classes OOPL and PPL, from olive oil and pomace, respectively. This method has been successfully used on vegetable oils [4]. In order to evaluate the method's suitability concerning the recovery of PPL, [^3^H]-PAF was used. Pooled ethanol phases, of 750 mL total volume, yield 97% of polar lipids as evaluated with [^3^H]-PAF [28].

Olive oil polar lipids (OOPLs) and pomace polar lipids (PPLs) derived, using the modified counter current distribution, from olive oil and pomace, respectively, were weighted and subjected to chemical determinations. Chemical determinations for total sugars, esters, and phenolic compounds were positive, while phosphorus was not detected (Table 1). These results are in accordance with the ones already reported in our previous work [28]. GC fatty acid analysis performed on OOPL showed the existence of palmitic (16 : 0), palmitoleic (16 : 1), stearic (18 : 0), oleic (cis 18 : 1), linoleic (18 : 2), and *α*-linolenic (18 : 3) fatty acids, whereas in PPL palmitic(16 : 0), oleic (cis 18 : 1), and *α*-linolenic (18 : 3) fatty acids were detected. The results expressed as (%) of the total OOPL and PPL are presented in Table 1. The above extracts were tested in vitro for inhibition of PAF-induced platelet aggregation. Both OOPL and PPL exhibited antithrombotic activity. The aforementioned inhibitory activity was retained for a wide range of tested amounts ranging from 0.01 ng to 1.12 *μ*g. The most diluted concentrations of OOPL and PPL that resulted in 50% inhibition of PAF activity were 1.5(±0.009) × 10^−10^ M and 1.1(±0.004) × 10^−10^ M, respectively, based on sugar determination. Final concentrations are given based on sugar determination since the bioactive microconstituents from both extracts have been characterized as glycolipids [4, 28]. Statistical analysis showed that OOPL IC_50_ value was higher (*P* < .05) than PPL IC_50_ value.

Consequently, [^3^H] PAF binding assay on washed rabbit platelets was studied in both absence and presence of unlabelled PAF as well as in the presence of OOPL, PPL, and BN52021 as a reference compound.

Scatchard analysis of binding data indicates the presence of two populations of binding sites: one with high affinity (specific binding) with an equilibrium dissociation constant Kd = 2.75 ± 1.16 nM and a maximum number of binding sites Bmax = 391 ± 112 fmol/10^8^ platelets (2355 ± 674 sites per platelet) and another one with low affinity (nonspecific binding) with an equilibrium dissociation constant Kd = 8.01 ± 2.47 nM and a maximum number of binding sites Bmax = 739 ± 122 fmol/10^8^ platelets (4450 ± 735 sites per platelet).

Our results show that OOPL and PPL inhibit specific PAF binding on rabbit platelets. The specific PAF receptor antagonist, BN 52021, inhibited PAF binding on rabbit platelets at a concentration of 2.3(±0.8) × 10^−7^ M. In the same way, the most diluted concentrations of OOPL and PPL that resulted in 50% inhibition of PAF binding were 1.5(±0.2) × 10^−7^ M and 0.42(±0.11) × 10^−7^ M, respectively, based on sugar determination.

Rabbit weight at the beginning (0 days) and at the end (22 days) of the experiment, as well as the daily food consumption, is presented in Table 2. Body weight in each group was significantly increased at the end of the experimental time (*P* < .05). No statistical difference was detected in rabbit weight among groups in both time intervals. In addition, all groups consumed equal amounts of food (*P* > .05).

At the end of the experimental time plasma TC, HDL-C, LDL-C levels were significantly increased in all rabbits, while TG levels in plasma were significantly increased only in group A (Table 3).

At the end of the experimental time, EC_50_ values were significantly increased only in groups B and C, while in group A these values were significantly decreased, compared to those of the initial time.

Significant differences were also observed in EC_50_ values of groups B and C compared to those of group A (Table 4). PAF levels, both free and bound, measured in rabbit blood and plasma PAF-AH activity were significantly elevated (*P* < .05) at the end of the experimental time (22 days) in all groups (Table 4).

The present study was focused on the early steps of atherogenesis, characterized by foam cell formation, where PAF, as the most active inflammatory lipid mediator, is believed to play critical role. The early atherosclerosis lesions, which were observed as foam cell layers and assessed as thickness and surface area developed inside the blood vessels, are presented in Table 5.

**Figure 2:**
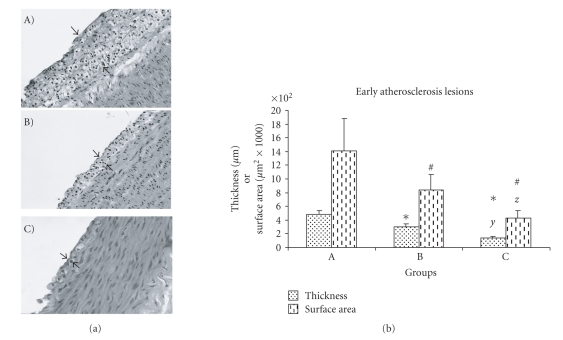
(a) Optic microphotographs (×200 for A and B; ×400 for C) of representative atherosclerosis lesions of aortic wall sections stained with haematoxylin and eosin. The arrows indicate the observed atherosclerosis lesions. A: atherogenic diet, B: atherogenic diet enriched with OOPL, C: atherogenic diet enriched with PPL. (b) Thickness and surface area of early atherosclerosis lesions of the three experimental groups, expressed as mean ± sd; *Significant difference for groups B and C versus group A in atherosclerosis lesion thickness (*P* < .05). ^#^Significant difference for groups B and C versus group A in atherosclerosis lesion area (*P* < .05). *y, z* Significant difference for group C versus group B in atherosclerosis lesion thickness and area respectively (*P* < .005). Statistical analysis was based on independent sample *t*-test.

Representative pictures of the morphometric analysis performed on the aorta specimens appear in Figure 2, where atherosclerosis lesions (foam cell layers) are indicated with arrows. Statistical analysis on the morphometric assessment data concerning thickness and surface of early atherosclerosis lesions in groups revealed that rabbits consumed atherogenic diet enriched in OOPL or PPL (groups B and C, resp.) developed lower early atherosclerosis lesions than rabbits consumed atherogenic diet only (Group A) (*P* < .05). Moreover, the atherosclerosis lesions in group C were also lower than those noted in group B (*P* < .05).

## 4. DISCUSSION

Olive oil, over the years, has been the subject of many in vivo studies, concerning its antiatherosclerotic effect. De la Cruz et al. reported that virgin olive oil administration to cholesterol-fed rabbits reduced vascular thrombogenicity of the subendothelium, increased antithrombotic activity in the endothelium, and decreased foam cell nuclear count and percentage of the wall occupied by foam cells [7]. Aguilera et al. showed that olive oil administration on atherosclerotic rabbits, although does not regress the phenomenon, is able to stop further the progression of atherosclerosis process [41]. The beneficial effects of olive oil were initially attributed to oleic acid and MUFAs, in general, through their action as antioxidants. Recent studies reported that olive oil anti-atherosclerotic effect might also be attributed to microconstituents of olive oil, such as phenolic compounds or phenolic glycosides [4, 5, 42].

PAF has been implicated in atherosclerosis [2, 15–18] while specific PAF-antagonists (BN 52021) as well as the ones extracted from olive oil inhibited atherosclerosis development on cholesterol fed rabbits [27, 43].

In the present study, such PAF-antagonists were extracted from both olive oil and pomace. The extraction process yielded 97% of pomace polar lipids as evaluated with [^3^H]-PAF that shows the method's suitability for solid samples. Total sugar determination showed that both extracts contain equal amount of sugars, expressed as glucose, and for this reason IC_50_ values expression was based on this determination. Phenolic compound determination showed that PPL contains almost twice the phenolic compound amount than OOPL. This was of no great surprise, since pomace has a higher concentration in olive skin and pigments, both rich in phenolic compounds [9–11], than olive oil. Concerning fatty acid composition, pomace oil has been reported to have pretty much the same fatty acid composition as olive oil. This was expected, since pomace retains olive oil, which is further extracted using hexane, thus producing pomace oil mainly containing pomace fatty acids [44]. In the present study, fatty acid concentrations in PPL were significantly lower than the respective ones in OOPL. In addition, palmitoleic, stearic, and linoleic fatty acids were only detected in OOPL. Palmitic acid can be found in both OOPL and PPL, in approximately the same concentration. On the other hand, oleic and *α*-linolenic acid concentrations in PPL are lower than the ones in OOPL.

In a recent work [28], olive pomace lipid extract has been purified on HPLC and the most potent PAF antagonist derived from this procedure was characterized as glycerylether-sn-2-acetyl glycolipid based on chemical determinations and ESMS analysis. This active compound shares similar biological activity, chromatographic behavior, and possibly structure to the one from olive oil polar lipids.

PPL inhibition potency is higher than that of OOPL as indicated by their IC_50_ values, which may be attributed to their higher phenolic compound concentration whose antithrombotic activity has already been reported [21, 45]. PAF binding assays performed on rabbit platelets showed that both polar lipid extracts are specific PAF receptor antagonists. In a similar way, PPL inhibition potency is higher than that of OOPL. The aforementioned results indicate that these extracts exert their inhibitory effect on PAF actions by blocking PAF receptor.

Enrichment of group B atherogenic diet with OOPL was based on previous studies [7, 27]. The amount of PPL, which enriched group C atherogenic diet, was equivalent to that of OOPL according to their IC_50_ values.

Conflicting results, concerning lipid profile of rabbits fed with atherogenic diet enriched in olive oil, exist in previous studies [7, 27]. Our findings are partly in accordance with the aforementioned ones. All these data indicate that lipid profile may supply valuable information on the total condition of experimental models; however, it cannot provide information on the atherosclerosis progression and blood vessel condition.

Platelet aggregation ability as indicated by the EC_50_ values decreased in groups B and C while increased in group A. No significant difference was observed in the EC_50_ values between groups B and C after food consumption. These findings suggest that PAF-antagonists present in OOPL and PPL may be bioavailable in rabbit blood where they can exhibit anti-PAF activity. Our findings are in accordance with those of previous studies, in which EC_50_ values increased only in rabbit groups that had consumed atherogenic diet enriched either in olive oil or olive oil polar lipids [7, 27].

PAF levels have been shown to be elevated in various inflammatory diseases, such as allergy, asthma, sepsis, trauma, shock, acute pancreatitis, and diabetes. In turn, PAF molecule can also promote oxidation, thus decisively contributing to atherosclerosis initiation and progression, as has been proposed by our group [2]. In this study, levels of PAF, both the one loosely bound to blood components (defined as “free”) as well as the one strongly bound to blood components and cell-associated (defined as “bound”), were significantly elevated in all three rabbit groups. It is well known that a variety of cells such as platelets, monocytes, macrophages, and so on biosynthesize PAF and PAF-like lipids under atherogenic conditions, as well as the produced PAF and PAF-like lipids amplify cell activation, which in turn further increases PAF levels [2, 15, 46, 47].

PAF-AH is the key enzyme to PAF degradation and its activity significantly increases during this study in all three groups. The observed elevation may be attributed to the increased PAF levels since inflammatory mediators in vivo induce PAF-AH activity and PAF stimulates enzyme expression [48]. PAF-AH is suggested to serve either as a protective enzyme against acute inflammatory and oxidative factors, such as PAF, or as a marker, concerning both disease progression and severity, in long term oxidative stress conditions [49, 50]. In addition, overexpression of PAF-AH appears to induce anti-atherogenic properties in animal model [15].

Rabbits belonging to groups B and C developed early atherosclerosis lesions, at significantly lower degree, compared to group A. This is in accordance with our previous study in which the antiatherosclerotic effect of olive oil on cholesterol fed rabbits was attributed to its polar lipid extract (OOPL) [27]. Significant differences were also noted in the early atherosclerosis lesions of group C compared to those of group B, indicating that PPL exhibit its anti-atherosclerotic activity by blocking PAF receptor.

The specific antagonist of PAF, BN 52021, has been previously shown to exert beneficial effects against atherosclerosis development in rabbits [43]. In the present study, atherogenic diet enriched in either OOPL or PPL, which are specific PAF antagonists, causes inhibition of early atherosclerosis development in rabbits despite the fact that PAF levels are elevated in their blood. These findings suggest that PAF-antagonists from olive oil and olive pomace are able to slow down the atherosclerotic process at least at its early stages by inhibiting PAF binding and activity even in cases where PAF levels are highly increased.

In conclusion, olive pomace contains PAF specific antagonists with similar bioactivities to those of olive oil. These bioactive microconstituents inhibit both specific PAF binding and PAF activity (in vitro and in vivo), consequently inhibiting early atherosclerosis development. The above data reinforce the beneficial effect of food origin PAF antagonists against atherosclerosis development and indicate a main role of PAF in atherosclerosis.

## Figures and Tables

**Table 1:** Percentage yield, chemical determination, and fatty acid analysis results on OOPL and PPL.

	OOPL (*n* = 3)	PPL (*n* = 3)

Weight (mg/g olive oil)	12.09 ± 0.10	—
Weight (mg/g pomace)	—	31.45 ± 6.68
Yield (% w/w)	1.20 ± 0.01	3.14 ± 0.66
Esters (*μ*mol/mg polar lipids)	1.38 ± 0.03	0.50 ± 0.01
Sugars expressed as glucose (*μ*mol/mg polar lipids)	0.45 ± 0.01	0.44 ± 0.01
Phenolics expressed as gallic acid (*μ*mol/mg polar lipids)	0.030 ± 0.001	0.050 ± 0.001
Phosphorus (*μ*mol/mg polar lipids)	n.d.	n.d.
16 : 0 (mg/100 mg polar lipids)	0.125 ± 0.004	0.120 ± 0.003
16 : 1 (mg/100 mg polar lipids)	0.041 ± 0.001	n.d.
18 : 0 (mg/100 mg polar lipids)	0.008 ± 0.004	n.d.
cis 18 : 1 (mg/100 mg polar lipids)	1.039 ± 0.022	0.608 ± 0.022
18 : 2 (mg/100 mg polar lipids)	0.122 ± 0.003	n.d.
18 : 3 (*n* − 3) (mg/100 mg polar lipids)	0.094 ± 0.005	0.038 ± 0.009

OOPL: olive oil polar lipid; PPL: Pomace Polar Lipids; n.d.: not detected; Values are expressed as mean ± sd.

**Table 2:** Body weight, daily food consumption and diet composition of experimental groups.

	Group A	Group B	Group C

Diet composition (% w/w)	Cholesterol (1.00%)	Cholesterol (1.00%) + OOPL (0.16 %)	Cholesterol (1.00%) + PPL (0.13%)
Body weight at 0 days (g)	2943 ± 137	2868 ± 172	2977 ± 102
Body weight at 22 days (g)	3383 ± 140*	3298 ± 220*	3438 ± 148*
Body weight increase (g)	440 ± 113	430 ± 130	461 ± 144
Daily food consumption (g)	166.3 ± 8.2	164.0 ± 10.6	159.3 ± 3.8

OOPL: Olive Oil Polar Lipids; PPL: Pomace Polar Lipids. Values are expressed as mean ± sd. *Significant difference for *P* < .05 within the same group, 22 days compared to 0 days, according to paired sample *t*-test; no statistical difference was detected in rabbit weight among groups both at 0 and 22 days.

**Table 3:** Lipid profile of experimental groups.

		Group A	Group B	Group C

Total cholesterol (mg/dL)	0 days	44	53.75	48
		(36.50–50.50)	(48.62–63.75)	(41.38–50)
	22 days	1348*	1421*	1054*
		(1199–1378)	(782.6–1703)	(775.0–1702)

HDL Cholesterol (mg/dL)	0 days	22.0	32.00	25.50
		(19.50–24.50)	(28.12–39.38)	(21.12–27.62)
	22 days	81.25*	77.50*	33.75*,**,^#^
		(65.88–98.25)	(58.25–89.50)	(28.50–43.88)

LDL Cholesterol (mg/dL)	0 days	11.50	14.05	13.40
		(8.650–19.30)	(12.75–18.98)	(10.98–17.35)
	22 days	1195*	1332*	1003*
		(1106–1242)	(696.1–1592)	(730.0–1616)

Triglyceride (mg/dL)	0 days	81.5	75.5	79.25
		(75.75–92.5)	(72.50–96.25)	(61.62–103.5)
	22 days	220.5*	102.2	97.25**
		(110.6–285.9)	(64.25–164.12)	(82.75–146.9)

A: atherogenic diet, B: atherogenic diet with OOPL, and C: atherogenic diet with PPL. Values are expressed as median (25th percentile-75th percentile) (*n* = 6). *Significant difference for *P* < .05 within the same group, 22 days compared to 0 days, according to Wilcoxon test. **Significant difference for *P* < .05 compared to group A, according to Mann-Whitney *U*-test; ^#^Significant difference for *P* < .05 compared to group B, according to Mann-Whitney *U*-test.

**Table 4:** Biochemical parameters of experimental groups.

	Day	Group A	Group B	Group C

Free PAF (pM)	0	15.7	7.26	5.84
		(13.5–19.9)	(6.73–14.4)	(3.92–10.8)
	22	36.9*	34.5*	21.8*
		(21.9–59.2)	(18.7–48.4)	(13.6–58.4)

Bound PAF (pM)	0	40.9	44.7	5.85
		(33.0–48.5)	(21.7–67.5)	(2.69–21.6)
	22	69.8*	59.6*	36.2*,**,^#^
		(50.4–75.4)	(42.5–80.2)	(18.2–38.4)

EC_50_ (nM)	0	31.5	22.5	24.5
		(22.9–42.0)	(14.8–41.2)	(19.0–41.2)
	22	12*	38.5*,**	38*,**
		(11.8–16.2)	(20.5–67.5)	(23.0–93.5)

PAF-AH activity [pmolPAF/(min × *μ*L PPP)]	0	124	224	181
		(93.9–143)	(180–247)	(160–209)
	22	262*	333*	288*
		(228–305)	(284–399)	(215–312)

A: atherogenic diet, B: atherogenic diet with OOPL, and C: atherogenic diet with PPL. Values are expressed as median (25th percentile-75th percentile) (*n* = 6). *Significant difference for *P* < .05 within the same group, compared to 0 days, according to Wilcoxon test. **Significant difference for *P* < .05 compared to group A, according to Mann-Whitney *U*-test. ^#^Significant difference for *P* < .05 compared to group B, according to Mann-Whitney *U*-test.

**Table 5:** Assessment of early atherosclerosis lesions observed in rabbit aortas.

Early atherosclerosis lesions evaluation				
Groups	Thickness (*μ*m)	Surface area (*μ*m^2^) × 1000

A	489 ± 49	1408 ± 470
B	298 ± 49*	835 ± 234^#^
C	145 ± 20*,^*y*^	429 ± 105^#,*y*^

Values of early atherosclerosis thickness and surface area occupied by foam cells are expressed as mean ± sd. *Significant difference for groups B or C versus group A in atherosclerosis lesion thickness (*P* < .05). ^#^Significant difference for groups B or C versus group A in atherosclerosis lesion surface area (*P* < .05). ^*y*^Significant difference for group C versus group B in atherosclerosis lesion thickness and surface area, respectively, (*P* < .005). Statistical analysis was based on independent sample *t*-test.
